# Implementing shared decision making for early-stage breast cancer treatment using a coproduction learning collaborative: the SHAIR Collaborative protocol

**DOI:** 10.1186/s43058-023-00453-z

**Published:** 2023-07-14

**Authors:** Danielle Schubbe, Renata W. Yen, Hannah Leavitt, Rachel C. Forcino, Christopher Jacobs, Erica B. Friedman, Maureen McEvoy, Kari M. Rosenkranz, Kristin E. Rojas, Ann Bradley, Eloise Crayton, Sherrill Jackson, Myrtle Mitchell, A. James O’Malley, Mary Politi, Anna N. A. Tosteson, Sandra L. Wong, Julie Margenthaler, Marie-Anne Durand, Glyn Elwyn

**Affiliations:** 1 The Dartmouth Institute for Health Policy and Clinical Practice, Geisel School of Medicine at Dartmouth College, Lebanon, NH 03756 USA; 2 Department of Surgery, New York University Langone Health, Bellevue Hospital, New York, NY 10016 USA; 3 Breast Surgery Division, Department of Surgery, Montefiore Medical Center, Montefiore Einstein Center for Cancer Care, Bronx, NY 10467 USA; 4 Department of Surgery, Dartmouth-Hitchcock Medical Center, Lebanon, NH 03756 USA; 5 Dewitt-Daughtry Department of Surgery, University of Miami Miller School of Medicine, Miami, FL 33136 USA; 6 Breakfast Club, Florissant, MO 63032 USA; 7 Division of Public Health Sciences, Department of Surgery, School of Medicine, Washington University in St. Louis, St. Louis, MO 63110 USA; 8 Department of Surgery, Washington University in St. Louis, St. Louis, MO 63110 USA; 9 Centre Universitaire de Médecine Générale Et Santé Publique, Unisanté, Rue du Bugnon 44, CH-1011 Lausanne, Switzerland; 10 UMR 1295, CERPOP, Université de Toulouse, Université Toulouse III Paul Sabatier, Toulouse, France

## Abstract

**Background:**

Shared decision making (SDM) in breast cancer care improves outcomes, but it is not routinely implemented. Results from the What Matters Most trial demonstrated that early-stage breast cancer surgery conversation aids, when used by surgeons after brief training, improved SDM and patient-reported outcomes. Trial surgeons and patients both encouraged using the conversation aids in routine care. We will develop and evaluate an online learning collaborative, called the SHared decision making Adoption Implementation Resource (SHAIR) Collaborative, to promote early-stage breast cancer surgery SDM by implementing the conversation aids into routine preoperative care. Learning collaboratives are known to be effective for quality improvement in clinical care, but no breast cancer learning collaborative currently exists.

Our specific aims are to (1) provide the SHAIR Collaborative resources to clinical sites to use with eligible patients, (2) examine the relationship between the use of the SHAIR Collaborative resources and patient reach, and (3) promote the emergence of a sustained learning collaborative in this clinical field, building on a partnership with the American Society of Breast Surgeons (ASBrS).

**Methods:**

We will conduct a two-phased implementation project: phase 1 pilot at five sites and phase 2 scale up at up to an additional 32 clinical sites across North America. The SHAIR Collaborative online platform will offer free access to conversation aids, training videos, electronic health record and patient portal integration guidance, a feedback dashboard, webinars, support center, and forum. We will use RE-AIM for data collection and evaluation. Our primary outcome is patient reach. Secondary data will include (1) patient-reported data from an optional, anonymous online survey, (2) number of active sites and interviews with site champions, (3) Normalization MeAsure Development questionnaire data from phase 1 sites, adaptations data utilizing the Framework for Reporting Adaptations and Modifications-Extended/-Implementation Strategies, and tracking implementation facilitating factors, and (4) progress on sustainability strategy and plans with ASBrS.

**Discussion:**

The SHAIR Collaborative will reach early-stage breast cancer patients across North America, evaluate patient-reported outcomes, engage up to 37 active sites, and potentially inform engagement factors affecting implementation success and may be sustained by ASBrS.

**Supplementary Information:**

The online version contains supplementary material available at 10.1186/s43058-023-00453-z.

Contributions to the literature

• The SHAIR Collaborative is a novel breast cancer learning collaborative engaging breast cancer care teams across North America in implementing shared decision making by using two variations of an early-stage breast cancer conversation aid.
• Our evaluation plan, guided by the Reach Effectiveness-Adoption Implementation Maintenance framework, uses multiple established frameworks and will be useful for informing other implementation efforts.
• Implementation of the SHAIR Collaborative is guided by a community-based participatory research approach, involving all stakeholders in an effort to promote sustainability.

## Background

Early-stage breast cancer surgery treatment is preference-sensitive and therefore warrants shared decision making [1]. However, only 44 to 51% of patients with early-stage breast cancer achieve the degree of participation in decision making they desire, and many report poor knowledge about breast cancer surgery options [2–7]. Shared decision making in breast cancer care is important and improves patient outcomes like knowledge and decision regret, but it is not routinely implemented due to barriers like lack of appropriate training, access to evidence-based conversation aids, and the potential to disrupt workflows [8–10]. Patients and clinical teams alike desire sustained implementation of shared decision making in breast cancer care [9, 11].

The What Matters Most randomized controlled trial provided early-stage breast cancer patients and their surgeons with either a text-based or picture-enhanced with simpler words encounter-based conversation aid (Option Grid™ or Picture Option Grid) to facilitate shared decision making for this surgery decision [12]. The results of the What Matters Most trial demonstrate that the conversation aids, when used by surgeons after a brief training, led to significant increases in observed and patient-reported shared decision making, and improved patient-reported outcomes, including higher knowledge, lower decision regret, and perception of receiving more coordinated care [12, 13]. Picture Option Grid, as predicted, had a stronger impact among patients with lower health literacy and lower socioeconomic position. Interviews with What Matters Most surgeons and patients showed that both would encourage and promote the use of the conversation aids in future breast cancer care [11]. Intervention surgeons reported that the conversation aids did not lengthen consultation time and enhanced their usual care after using it a few times [11]. One review and other studies that have evaluated Option Grids have reported similar results [14–17]. Further evidence has recently been published showing the superiority of brief encounter-based tools [18].

Given that Option Grid and Picture Option Grid for early-stage breast cancer improve patient-reported outcomes and the conversation aids are desired in breast cancer care, we are implementing a shared decision making strategy that was formally tested and demonstrated effectiveness in the context of the What Matters Most trial [11, 12]. The effective shared decision making strategy included the two conversation aids and a brief training module for participating breast surgeons. In this implementation protocol, we augment the strategy by embedding it in an online learning collaborative. The learning collaborative approach is based on Wenger’s community of practice model and has been frequently adopted in healthcare quality improvement programs [19]. There is a well-established body of evidence to support the use of learning collaboratives, and a systematic review found that these types of collaboratives lead to significant improvements in clinical processes and patient outcomes [20, 21]. The SHared decision making Adoption and Implementation Resource (SHAIR) Collaborative will be hosted on an open-access website for breast surgeons and will include (1) free access to the early-stage breast cancer Option Grid and Picture Option Grid conversation aids in multiple languages, (2) brief shared decision making training, (3) implementation support through online and recorded webinars, implementation checklists, electronic health record and telehealth integration guidance, and other appropriate support materials, and (4) a feedback dashboard to anonymously share patient-reported outcomes from participating sites [22, 23].

Our specific aims are to (1) provide the SHAIR Collaborative and all of its resources to clinical sites and its eligible patients, (2) examine the relationship between access and use of the SHAIR Collaborative offerings and the success of the implementation, and (3) create a sustained learning collaborative in this clinical field to support ongoing use of shared decision making.

## Design and methods

All title/abstract, introduction, and methods have been reviewed utilizing StaRI, the Standards for Reporting Implementation studies (Additional file 1: Appendix 1) [24].

### Design and theoretical basis

Our theoretical basis is Rogers’ Diffusion of Innovation Theory [25]. Diffusion occurs through a five-step process, including knowledge/awareness of the innovation (i.e., early-stage breast cancer conversation aids), persuasion, decision to innovate, implementation of the innovation, and continuation of the innovation. The social system is a key element of diffusion of innovation [25, 26]. The development of an online learning collaborative will provide a foundation to support this element. We will target “early adopters”, but overall rely on developing an “early majority” of adopters, as those who adopt innovations sooner than most, but require evidence of effectiveness prior to adoption. We are targeting several stages of adoption with the learning collaborative: (1) dissemination of shared decision making utilizing evidence-based conversation aids, (2) implementation of the conversation aids in diverse clinics across North America by utilizing an online learning collaborative approach, and (3) potential sustainability/continuation of the learning collaborative [27].

Since the text-based and picture-enhanced conversation aids were found to be effective in improving shared decision making compared to usual care in a comparative effectiveness trial, we outline below our design for an implementation project to broadly implement the conversation aids.

We will conduct a two-phased implementation project: phase 1 is to pilot our implementation approach, and phase 2 is to scale up the implementation. In phase 1, we will design and develop the implementation platform in conjunction with our project stakeholders. We will then test the implementation platform and approach at our phase 1 pilot sites across the USA, who are our “early adopters” (four out of five phase 1 sites were participating sites in the comparative effectiveness trial). Next, we will refine the approach and prepare for phase 2. In phase 2, we will broadly implement the platform in additional clinical sites across the USA, Mexico, and Canada. Phase 2 sites are the “early majority” of adopters and will learn from phase 1 sites’ “early adopters”. Throughout both phases, we will explore features of sustainable implementation. These sites and our stakeholders will become the SHAIR Collaborative, an online learning collaborative committed to implementing shared decision making for patients with early-stage breast cancer.

### Settings

Phase 1 settings will include five academic medical centers: Dartmouth-Hitchcock Medical Center (Lebanon, NH), Washington University in St. Louis (St. Louis, MO), Montefiore Medical Center (Bronx, NY), Bellevue Hospital (New York, NY), and the University of Miami (Miami, FL). We selected these sites for initial testing of the implementation platform to ensure broad representation of patient demographics and underlying interest in implementing shared decision making in their practices. Four of the five phase 1 sites participated in the What Matters Most trial [12].

Phase 2 settings will include up to an additional 32 clinical sites: single clinician private practices, multi-clinician private practices, local and regional hospitals, and academic medical centers. Phase 2 sites will be located in North America. We recruited an initial set of phase 2 sites when applying for funding and will finalize the set of sites prior to the start of phase 2. Sites will be eligible if they have practicing breast cancer surgeons who treat patients with early-stage breast cancer. Phase 2 sites will receive a small honorarium to cover costs associated with the minor research activities of participation in the SHAIR Collaborative. We will allow for additional sites to join the SHAIR Collaborative as feasible.

### SHAIR Collaborative platform and offerings

In phase 1, we will design the implementation platform which will include key offerings, all centered around supporting implementation of shared decision making for early-stage breast cancer patients. The SHAIR offerings are based on our assessment of positive implementation factors reported to date from previous implementation experience [12, 28]. Adaptations to the resources/offerings and implementation strategy will be guided by project stakeholders and made as necessary. All offerings will be available via the open-access website.

1. *Free and direct access to the Option Grid conversation aids*. Produced by EBSCO Health, these conversation aids are continuously updated and offered in perpetuity free of charge [29]. A dedicated website will ensure direct open access to the Early-Stage Breast Cancer Surgery Option Grid and Picture Option Grid, as well as downloadable PDF versions in multiple languages (English, Spanish, Mandarin Chinese). We will obtain certified translations of the conversation aids for additional languages as requested. We will encourage the use of interpreters and interpretation technology where patients speak other languages that Option Grid is not currently offered in.
2. *Training and instructional videos*. We will develop a set of short training videos that will be designed to train clinicians in shared decision making and implementation and support the SHAIR Collaborative. We have planned for an initial set which includes an introduction to the SHAIR Collaborative, introduction to shared decision making, communicating shared decision making using the three-talk model [30], using Option Grids as an encounter-based conversation aid in clinical practice, and how to implement conversation aids in diverse clinical settings. This list of videos and deciding on future instructional videos will be refined and added to based on stakeholder input.
3. *Integration guides*. Our experience indicates that electronic health record (EHR) integration of conversation aids is complex, requiring clinical leadership, institutional prioritization, and funding for and access to EHR integration experts [28]. In collaboration with the expertise of EBSCO Health and the project’s other stakeholders, we will develop a downloadable PDF guide to integrating the Option Grids and Picture Option Grids into the EHR. Rapid uptake of telehealth as a result of the COVID-19 pandemic has transformed the use of patient portals to send materials to patients ahead of their encounters [23]. We will provide downloadable PDF guides on how to embed the conversation aids into workflow processes so that patients can be given the conversation aids at the appropriate time, allowing for context-sensitive delivery of the conversation aids.
4. *Feedback dashboard*. Phase 1 and phase 2 sites will provide monthly data on patient reach. We will also collect optional, anonymous online patient surveys from participating sites. A summary of these data will be displayed via a feedback dashboard.
5. *Webinars*. We will host live, recorded SHAIR Collaborative webinars starting in phase 2. The content, length, format, platform, and frequency of the webinars will be decided and refined by project stakeholders. Recordings and relevant materials from these webinars will be available to SHAIR Collaborative members.
6. *Support center and forum*. Members of the Collaborative will be able to issue support requests and engage with other Collaborative members via email and website support forum. There will also be a section of the website dedicated to frequently asked questions.

### Site onboarding and training

We will ask surgeons at each site to view the initial set of training videos available on the platform (component 2 above) before beginning implementation at their site. We will also offer virtual site initiation visits (SIVs) to all phase 1 and phase 2 sites but will only require them for phase 1 sites. We will invite surgeons, clinic and administrative staff, and any other appropriate clinical or non-clinical contacts to the SIVs based on site needs. At minimum, each SIV will include an introduction of SHAIR and training (if needed) and discussion regarding the site’s implementation strategy. Lastly, we will provide an implementation checklist to phase 2 sites based on guidance and best practices from phase 1 sites.

### Data collection and evaluation

We will use the RE-AIM framework to guide our data collection and evaluation of this implementation project. RE-AIM addresses reach, effectiveness, adoption, implementation, and maintenance [31].

#### Reach

##### Data collection

Our primary outcome is patient reach, which is defined as the number of patients who are given the opportunity to use one of the conversation aids over the number of eligible patients, by site. Surgeons using the conversation aids at each site will determine patient eligibility based on clinical judgment. We will determine patient reach for each site on a monthly basis using the following data:

1. Reported uses of the conversation aids—We will email each site monthly to request data and notes for the previous month regarding patient reach. Other uses of the conversation aids can include using paper copies, sending links or PDFs to patients by email, patient portal, EHR, or other distribution methods.
  From our experience, we anticipate that paper versions will be used only once with patients [11]. However, we realize a patient may be given multiple copies throughout their care and/or for others to view (family members, friends, caregivers). While this may occur, we do not anticipate our sites to regularly provide multiple paper copies of an Option Grid/Picture Option Grid. In the instance multiple copies are given, we will work with each site to try and get as accurate of count as possible.
2. Online access to conversation aids via platform—The SHAIR Collaborative website is able to track the downloads of the conversation aids or clicks out to the linked EBSCO website for the interactive tools. Downloads will be tracked and reported on a monthly basis.

##### Evaluation

Reported patient reach data and site-specific online accesses of the conversation aids will be counted for each site monthly. We will calculate patient reach as the percentage of eligible patients receiving the Option Grid or Picture Option Grid. We will report this overall and by site for sites with sufficient data. In the patient reach email response from sites, any details given regarding barriers and facilitators to patient reach will be noted and responded to, as appropriate.

#### Effectiveness

##### Data collection

Patient-level secondary outcomes include patient-reported shared decision making, assessed using the validated 3-item collaboRATE measure [32, 33], decisional conflict, assessed using the validated 4-item measure, SURE [34], integration of healthcare delivery, assessed using the validated 4-item measure, integRATE [35, 36], a self-developed question reporting the treatment chosen, and a one-item assessment of gist understanding from the validated Decision Quality Instrument for breast cancer [37].

We will utilize Qualtrics for patients to complete after their clinical encounter, if interested, at each site. This survey will be online, anonymous, and optional. The survey will include questions about the conversation aid, patient demographics, as well as the secondary outcomes listed above. The survey link and QR code will be available via postcards and/or clinic flyers given to patients or put in strategic locations in the clinic. We will offer a $20 USD gift card incentive to encourage the optional survey responses. Survey participants will be redirected to a separate survey to provide their email address and receive their gift card so that patient responses and email addresses remain separate.

##### Evaluation

Patient-level data from the optional, anonymous online survey will be clustered by site. Once a site has secured five survey responses, data will be collated and presented on the SHAIR Collaborative website’s feedback dashboard. Individual site data will be reported to sites, at request.

#### Adoption

##### Data collection

Our main measure of adoption is the total number of SHAIR Collaborative sites that actively participate. Sites that are interested in joining the SHAIR Collaborative will sign a letter of support. We will note when sites join the SHAIR Collaborative and if they become inactive (based on lack of responsiveness or signaling their resignation). We will recruit additional sites routinely throughout the project guided by stakeholder feedback.

Additionally, we will conduct brief interviews with SHAIR site champions (clinical and non-clinical) approximately 1 year into implementation and again towards the end of the formal implementation period before sustainability. The interview guide will be designed using Diffusion of Innovation and other relevant frameworks [25]. Interviews will be first conducted with phase 1 sites and then phase 2 sites, as relevant.

Lastly, we will measure social interconnectedness within SHAIR Collaborative over time utilizing quarterly online surveys to active SHAIR Collaborative members. The surveys will include identifying names and the number of contacts made within the Collaborative [38].

##### Evaluation

We will report the number of active SHAIR sites and the number of those that became inactive at the conclusion of the project funding period. We will also report the proportion of all sites that join the SHAIR Collaborative and are still active at the conclusion of the project funding period.

The site champion interviews will be conducted using an interview guide designed in collaboration with project stakeholders and informed by implementation progress. The interviews will be audio-recorded and conducted by one team member while another team member will take notes during each interview. Two independent reviewers will review the interviews and conduct qualitative analysis. A third individual will be available to arbitrate, if necessary.

We will use regression analysis to assess the impact of social interconnectedness within the collaborative over time on the primary outcome of site-level patient reach [39].

#### Implementation

##### Data collection

To assess the extent to which the conversation aids are normalized at phase 1 sites, we will use the unadapted Normalization MeAsure Development (NoMAD) instrument [40]. NoMAD is a 23-item questionnaire designed to evaluate implementation processes according to Normalization Process Theory [41]. Our primary contact at each phase 1 site will identify eligible individuals and provide them with an anonymous Qualtrics link to survey. The survey will include an information sheet, three questions about the respondent’s site, role, and years of experience, and then each section of NoMAD. We will ask our primary contacts to include clinical and non-clinical roles that may be involved in the implementation of the conversation aids. We will ask them to distribute the NoMAD survey three times: prior to implementation, halfway through the implementation project period, and at the end of the implementation project period.

We will use an expanded framework for reporting adaptations and modifications to evidence-based interventions (FRAME) to document changes to the early-stage breast cancer conversation aids [42]. We will use a framework for documenting modifications to implementation strategies in healthcare (FRAME-IS) to document changes to our implementation strategy [43].

We will collect a range of SHAIR Collaborative and implementation facilitating factors on a monthly and quarterly basis (see Table 1). Each factor is based on our implementation approach and will be assessed based on how often sites engage in each factor. We will complete assessments as a team using logs from the month or quarter to input the values for that time period for each site.

**Table 1:** List of implementation facilitating factors

Factor	Rating approach	Notes/scoring
**Monthly**		
Communications engagement	Yes/no	**• Yes** = communicated with us at least 1 in a given month (e.g., emailed back with patient uses) **• No** = did not communicate with us in a given month
Dashboard development	Yes/no	**• Yes** = Pt surveys collected **• No** = Pt surveys not collected
Website engagement	Yes/no	**• Yes** = downloaded something on website or engaged via comments on website **• No** = did none of those
Other notes on site’s engagement	N/A	
**Quarterly**		
Clinical champion engagement	High/intermediate/low	For example, **• Low** = lack of interest; **• High** = Evident interest and support
Team engagement	High/intermediate/low	For example, **• High** = attendance of team members at meetings **• Low** = no attendance of team members at meetings
EHR/telehealth/patient portal Integration progress	High/intermediate/low	For example, **• High** = engaging in at least one integration **• Intermediate** = interested by not complete **• Low** = not happening

##### Evaluation

We will calculate descriptive statistics summarizing the NoMAD survey results for each phase 1 site at the conclusion of the implementation period.

Separately, we will use the factors shown from Table 1 as variables in a linear regression analysis to explore which factors might be associated with high levels of patient reach (clinical championship, team engagement, communications engagement etc.) and therefore might predict future patient reach. For these regressions, patient reach will be the dependent variable and each implementation factor will be an independent variable. We will assess each item at the site level so that “site” will be the unit of analysis. We will control for patient demographics and other potential sources of site heterogeneity, if possible.

Additionally, we will conduct mixed-effects linear or logistic regression analyses to examine the association between these implementation factors and our patient-reported outcomes. We will conduct an analysis for each outcome while controlling for patient demographics as fixed effects. For this analysis, “patient” will be the unit of analysis but we will account for clustering at the site level as a random effect.

#### Maintenance

##### Data collection

During the project funding application, we developed a partnership with the American Society of Breast Surgeons (ASBrS) to initially recruit interested clinical sites and then serve as a willing candidate to sustain the SHAIR Collaborative and/or components of the SHAIR Collaborative. We will communicate regularly with dedicated team members from ASBrS to work on the sustainability strategy and all meetings will be documented by a project team member. One core promoter of sustainability is the open-access nature of the early-stage breast cancer Option Grids on the EBSCO website. Another will be the open-access online learning collaborative platform.

##### Evaluation

We will report our progress on sustainability plans with ASBrS at the conclusion of the project funding period.

### Data management

Patient reach data and implementation facilitating data will be tracked via spreadsheets and stored in a shared project folder. Survey data will be stored in the Dartmouth Qualtrics database, a HIPAA-compliant web-based data management system. Dartmouth project staff will not have access to the EHRs at any participating phase 1 or phase 2 sites. Our database management system is designed to comply with the ICH Good Clinical Practice guidelines. For statistical analysis, data will be downloaded and stored on an encrypted hard drive owned by Dartmouth College.

### Project management

We will use a community-based participatory research approach to ensure lasting and valuable partnerships with our variety of project stakeholders (clinical, research, patient, community, and organizational) [44–47].

We will hold quarterly steering group meetings with all key personnel and additional project members who advise on the project’s scientific and strategic direction using Zoom conferencing. The duties of the steering group will include supervising and monitoring study progress and providing feedback.

We will hold quarterly Community Advisory Board meetings that include all patient and stakeholder partners using Zoom audio conferencing. In these meetings, we examine project progress and materials and discuss relevant project items with a specific focus on the community perspective.

We will regularly engage with our phase 1 and phase 2 site contacts through various communication strategies guided by levels of engagement and feedback from the clinicians and other stakeholders. It is important to tailor communication methods to accommodate the busy schedules of clinicians and their teams. We will note these various communication strategies and their progression to determine which may be best for sustainability.

## Discussion

We are implementing evidence-based strategies for shared decision making about early-stage breast cancer surgery by creating and evaluating an online learning collaborative, the SHAIR Collaborative. The SHAIR Collaborative will be a group of clinical sites across North America implementing shared decision making by using two early-stage breast cancer conversation aids, called Option Grids. The SHAIR Collaborative will (1) reach early-stage breast cancer patients across diverse clinics in North America, (2) evaluate and visualize patient-reported outcomes, (3) contain up to 32 actively participating sites, (4) inform which factors of engagement affect implementation success (patient reach), and (5) potentially be sustained by the American Society of Breast Surgeons (ASBrS).

Early-stage breast cancer patients are not often involved in the decision making for their surgery to the extent they prefer, and patients and surgeons alike wish to have effective conversation aids, like the early-stage breast cancer Option Grid, implemented in future breast cancer care [2–7, 11]. Dissemination will take place across diverse clinical settings in North America. Scalability of the SHAIR Collaborative is an important factor to consider throughout dissemination and implementation; an open-access website with an easy-to-use server-side and our collaboration with ASBrS on sustainability plans will support scalability. One major benefit for scalability and sustainability is that the conversation aids will be updated and offered for free in perpetuity, eliminating an important barrier to access (cost). Additionally, other engagement factors and utilization of SHAIR offerings may inform the sustainability strategy and other future implementation projects.

One practical limitation in this project is the challenge of engaging with busy clinicians. It is important to be willing to tailor the communication strategy to our sites in order to improve engagement. There is also a balance between under- and over-communicating, making it important to focus on the necessary items (especially pertaining to sustainability) to cover with clinicians. Another practical limitation is the generalized nature of the Option Grid conversation aids. Feedback regarding the statistical content on Option Grids is often site-specific, so we consistently suggest to use the conversation aids flexibly, allowing for adaptations for individual patients. One major strength of this project is the support from ASBrS, ensuring better chances at sustaining the whole or parts of the SHAIR Collaborative when the project funding is over. The support from our community-based partnerships, especially patient partners, provides a strong and diverse stakeholder group ensuring all voices are heard, which may help with sustainability. Another strength is the SHAIR Collaborative platform itself—open-access, completely online, and ability to support sites from across North America. We are also using well-established frameworks throughout the creation, data collection, and evaluation of the project: Diffusion of Innovation, RE-AIM, Normalization Process Theory, and FRAME and FRAME-IS [25, 31, 40, 42].

We will participate in numerous dissemination activities through conferences and other communication platforms. We will also engage all project stakeholders in the dissemination of our project findings.

## Supplementary Information

**Additional file 1.** 

## Data Availability

Not applicable.
